# Improved protein complex prediction with AlphaFold-multimer by denoising the MSA profile

**DOI:** 10.1371/journal.pcbi.1012253

**Published:** 2024-07-25

**Authors:** Patrick Bryant, Frank Noé

**Affiliations:** 1 Department of Mathematics and Informatics, Freie Universität Berlin, Germany; 2 The Department of Molecular Biosciences, The Wenner-Gren Institute, Stockholm University, Stockholm, Sweden; 3 Science for Life Laboratory, Solna, Sweden; 4 Microsoft Research AI4Science, Berlin, Germany; Hebrew University of Jerusalem, ISRAEL

## Abstract

Structure prediction of protein complexes has improved significantly with AlphaFold2 and AlphaFold-multimer (AFM), but only 60% of dimers are accurately predicted. Here, we learn a bias to the MSA representation that improves the predictions by performing gradient descent through the AFM network. We demonstrate the performance on seven difficult targets from CASP15 and increase the average MMscore to 0.76 compared to 0.63 with AFM. We evaluate the procedure on 487 protein complexes where AFM fails and obtain an increased success rate (MMscore>0.75) of 33% on these difficult targets. Our protocol, AFProfile, provides a way to direct predictions towards a defined target function guided by the MSA. We expect gradient descent over the MSA to be useful for different tasks.

## Author summary

AI networks can now predict the structure of protein complexes with high accuracy in the majority of cases. The accuracy of the predicted protein complexes is directly related to the quality of the input information. However, this information can be very noisy making the output of varying quality. An interesting finding is that AI networks used for structure prediction tend to know when wrong predictions are made based on confidence in the predictions themselves. Together, this suggests that one can look for more useful input information with the predicted confidence from the AI network. To improve the structure prediction of protein complexes, we here learn how to filter the input information so that AlphaFold-multimer can use it better based on the predicted confidence. We show that it is possible to do this efficiently and improve the structures in 33% of cases where AlphaFold-multimer struggles. The same filtering procedure can be used for other tasks as well, e.g. to search for alternative conformations although this remains to be studied.

## Introduction

Protein structure prediction of single chains is now highly accurate with AlphaFold2 (AF) [1]. The prediction of multimeric protein structures is not, as recent benchmarks report an average success rate (MMscore [2] above 0.75) of around 60% for dimers and the accuracy decreases with the number of chains [3]. By adapting the information of the multiple sequence alignment (MSA) that goes into AF, the network which is trained only on single chains can be adapted to outperform all other protein docking methods [4–6]. AlphaFold-multimer (AFM) uses the same adaptation of information but is trained in an end-to-end fashion obtaining slightly better performance [7].

Recycling significantly improves performance for both single- and multi-chain proteins [1,4]. In each recycling iteration, the input MSA is sampled to create input features (as the whole MSA can’t fit inside the network), embedding more information inside the network [7]. By making many predictions (6000), increasing the number of recycles and adding noise (activating dropout), the accuracy can go from poor to almost perfect in some cases [8–10]. Further, the confidence metrics provided through the predictions can separate the accurate cases from the inaccurate ones [9,11]. Together, this suggests that:

1. The information to make high-quality predictions is available in the MSAs.
2. The algorithm of AFM is sufficient to generate highly accurate structures given the correct information.
3. Accurate structures can be selected through quality metrics.

The main problem is therefore to find the right MSA information so that AFM can utilise it to generate high-quality predictions and further algorithmic advances may not be necessary. A procedure utilising similar concepts to generate MSAs for single-chain structures has been developed previously, although this was tested on only four single-chain structures [12].

The issue of how to find a combination of sequences with sufficient information to predict an accurate structure is difficult. During recycling, a combination of different samples of sequences taken from the MSA is built up. The cumulative representation resulting from this sampling, including the internal representation kept through the recycling, is what determines the outcome. As this is a complicated collection of information, it is unclear how to determine an optimal selection beforehand, which is why sampling is performed randomly [1,7].

A method to enhance the MSA information for the AFM network would potentially improve the accuracy of the protein complex predictions. As the quality metrics predicted by the network serve as a guide for model quality, we propose to use these to guide the representation of the MSA in the network to be more favourable. This idea is similar to advances in protein design which search for sequences that generate high confidence metrics predicted by AF [13–16]. The most efficient way to search for such a representation should be gradient-based, i.e. by backpropagating through AFM. By doing that, we suggest that the MSA profile is effectively denoised, similar to how a blurry image would be sharpened to become more clear.

## Results

### Improving the MSA profile with gradient descent

Structure prediction depends on coevolutionary information. Obtaining more ordered information greatly improves the performance of protein complex prediction [4]. The most important information that goes into AlphaFold-multimer (AFM) is the multiple sequence alignment (MSA) cluster profile. This is a statistical representation of which amino acids are likely to be found at certain positions in an MSA. The importance of the profile can be shown by setting a random MSA profile and keeping all other MSA features intact. The result is a completely disordered structure. If a structure can be destroyed by corrupting the profile, perhaps it can be improved by finding the most useful features in the evolutionary representation, i.e. *denoising it*.

**Fig 1:**
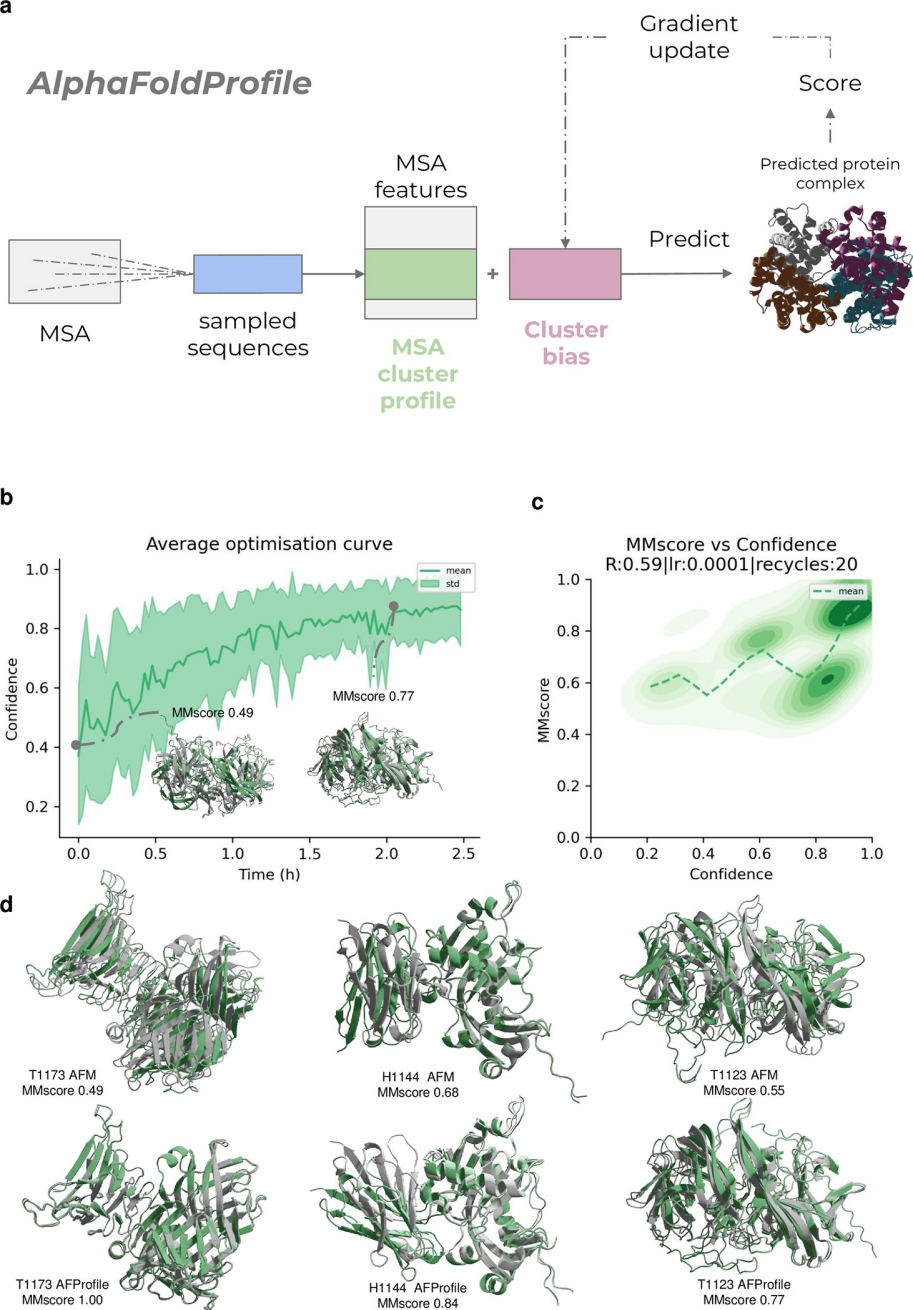
**a) The AFProfile Method.** Starting with MSAs generated by the default AlphaFold-multimer pipeline, sequences are sampled and MSA features are created. These features are used to predict the structure with AlphaFold-multimer and among the most important is the cluster profile. We learn a residual to this, the cluster bias, which we suggest effectively denoises the MSA profile into a representation that generates a higher confidence score and a more accurate structure. This can be seen as denoising a blurry image to make it sharper. **b)** Average predicted model confidence vs time in hours on one NVIDIA A100 GPU using gradient descent for the 7 CASP15 targets (H1134, T1123, T1173, H1141, H1144, H1140 and T1187). A learning rate (lr) of 1e-4 with the Adam optimiser and 20 recycles was used here (Methods). Example models for T1123 (green) are shown at different points of predicted confidence with the top-ranked CASP15 model in structural superposition (grey). In total, 100 optimisation steps were performed per target (n = 700). **c)** Confidence vs MMscore across the 7 CASP15 targets (H1134, T1123, T1173, H1141, H1144, H1140 and T1187). For each target, 100 optimisation steps were performed (n = 700). The Spearman R is 0.68, the lr 1e-4 and the number of recycles used set to 10 (Methods). A density plot using all samples (n = 700) and a running mean using a step of 0.05 confidence are shown. **d)** Examples from CASP15 with the best prediction in grey and AlphaFold-multimer(AFM) and AFProfile coloured green for targets H1144, T1123 and T1173. For T1173, the MMscore improves from 0.49 with AFM to perfect with AFProfile (1.0). For H1144, one of the chains is in the wrong orientation, while the right configuration is found with AFProfile (MMscore = 0.84). For T1123, both chains are slightly wrong (MMscore = 0.55), while AFProfile improves the score to 0.77 (accurate model >0.75).

In CASP15, one of the highest performing groups (Wallner [9], https://www.predictioncenter.org/casp15/zscores_multimer.cgi) achieved their performance by introducing noise into AFM and making many predictions (6000 in total) [9,17,18]. Although high performance was achieved in many cases, this procedure can likely not be deployed at scale due to the high computational requirement. Stopping criteria can be introduced to reduce the number of samples needed, but there is no guarantee that a good model will be the outcome as sampling a good MSA can be a rare event. To solve these issues we created a way to improve the MSA profile to obtain more accurate structures, a process we call **AFProfile** (Fig 1A).

AFProfile adds a bias to the MSA profile which acts as a correction term. The bias is created by performing gradient descent through the AFM network to maximise the confidence (Eq 1) AFM has in the predicted structure. The confidence that AFM predicts has been shown to correlate with model quality [9]. The process we propose to serve as a “denoising” of the MSA representation, similar to how a blurred image would be made clearer.

Confidence=0.8·iptm+0.2·ptm (1)

Here, iptm is the predicted TM-score [19] in the interface and ptm is that of the entire complex.

We optimised the procedure on seven targets from CASP15 in which the Wallner group outperformed the standard AFM prediction using AFsample (Methods). The bias addition being learned rapidly improves the confidence (Fig 1B) and we find that the confidence and MMscore trajectories correlate with a SpearmanR of 0.59 and a high density of structures with MMscores and confidences >0.8 (Fig 1C). AFM is outperformed with an average MMscore of 0.63 vs 0.76 for AFProfile (Table 1 and Fig 1C). For three targets (H1144, T1123 and T1173) the models go from unsuccessful with AFM to successful (MMscore>0.75) with AFProfile (Fig 1D).

**Table 1:** Prediction results on 7 targets from CASP15 from the Wallner group, AFM and AFProfile. The AFsample MMscores are higher on average, 0.97 vs 0.76 for AFProfile. Compared to AFM, the increase in MMscore is 0.13 on average (0.76 vs 0.64), making 3 additional targets successful (MMscore>0.75). The successful models (MMscore>0.75) are marked in bold.

Target	Reference MMscore (top model from CASP15)	AFsample MMscore	AF-multimer MMscore	AFProfile MMscore
H1134	**0.97**	**0.97**	**0.92**	**0.88**
H1140	**0.97**	**0.97**	0.62	0.61
H1141	**0.98**	**0.97**	0.68	0.68
H1144	**0.99**	**0.99**	0.68	**0.84**
T1123	**0.89**	**0.91**	0.55	**0.77**
T1173	**0.99**	**0.99**	0.49	**1.00**
T1187	**0.98**	**0.96**	0.49	0.51
Average	**0.97**	**0.97**	0.63	**0.76**

Compared to using AFsample, the performance of AFProfile is lower (average MMscores of 0.76 vs 0.97). Learning the residuals to the cluster bias speeds up the prediction at least 60-fold on average as 100 iterations are used compared to 6000 with AFsample. Since AFM v2.3 uses 100 models with 20 recycles [20] this process proves as efficient as the default AFM. Most importantly, AFProfile provides a way to direct the predictions towards more accurate complexes.

### Improving the success rate of AlphaFold-multimer on difficult targets

**Fig 2:**
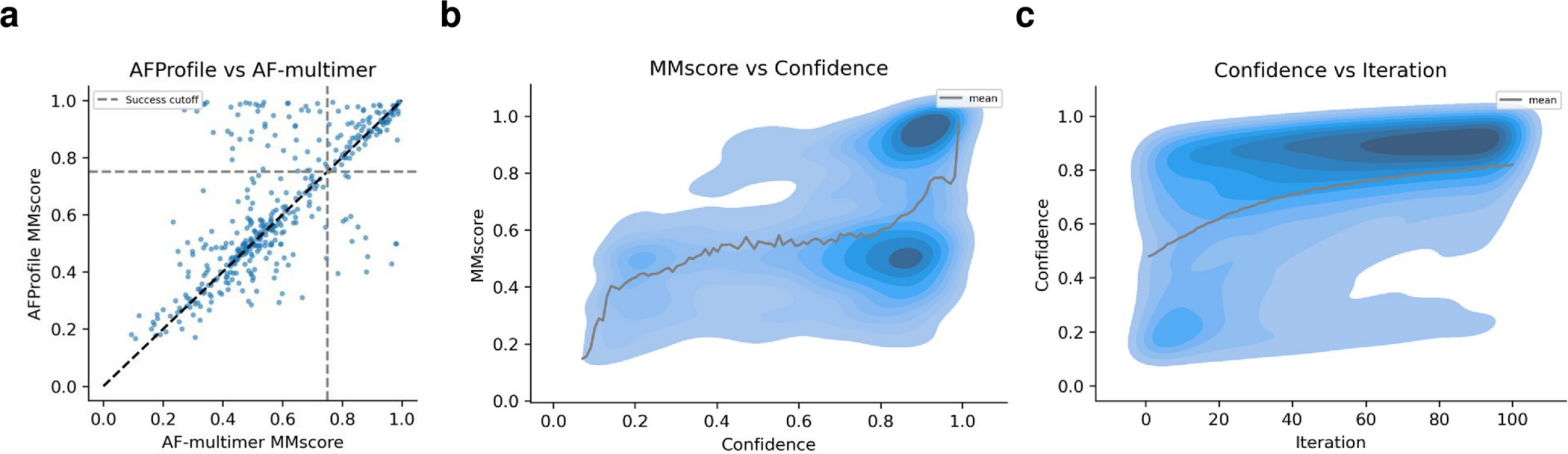
**a)** MMscores of difficult targets that have low ranking confidence (RC, Eq 1) (n = 487) with AF-multimer compared to AFProfile. In total, 33% of the failed examples can be rescued and selected (MMscore>0.75 and RC>0.8) with AFProfile. **b)** MMscore vs confidence. The density represents all predictions (100 per target, n = 48700) and the line is the running mean using a step size of 0.01 confidence. **c)** Density plot of the confidence vs iteration of gradient descent with AFProfile (n = 48700). At higher iterations, there is a strong density of high confidence. Following Fig 2B, this region is more likely to have high MMscores.

As we developed the learning of the MSA cluster bias on only 7 targets from CASP15, we now set out to evaluate the expected performance of AFProfile on a much larger set of 427 nonredundant complexes with 2–6 chains without homology to the training set of AFM [7]. These were selected from a recent benchmark of complexes as AFM reports low ranking confidence (RC) (<0.75, Eq 1), meaning that they can be selected from a single AFM run [1,3]. The success rate (MMscore>0.75 and RC>0.8) increases to 33% (139 complexes) for these difficult targets with AFProfile compared to 13% with AFM (Fig 2A).

Although the success rate increases to 33%, hidden failures emerge. For 200 complexes, the RC is >0.8 and the MMscore<0.75. Still, the RC is useful in selecting accurate predictions. Using the RC to select for accurate models (MMscore>0.75) we create a ROC-curve with a resulting AUC of 0.74 compared to 0.5 for a random selection (S1 Text). See S1 Text for USalign TM-scores (PearsonR = 0.996) [21].

A similar relationship between the MMscore and confidence as for the CASP15 set is observed. For confidences below 0.8, the average MMscore is 0.5 and only increases to 0.9 for confidences above 0.9 (Fig 2B). However, most complexes (71%) are not predicted accurately here resulting in a concentration of complexes with confidences of 0.8–0.9 and MMscores of 0.5, suggesting a higher difficulty in this set (Fig 2B). Fig 2C displays the relationship between the confidence and the iteration (gradient descent step). At higher iterations, there is a strong density of high confidence. Following Fig 2B, this region is more likely to have high MMscores. This suggests that the optimisation procedure does improve the outcome and that running iterations with AFP is favourable.

For some complexes, the optimisation procedure results in a lower MMscore (Fig 1A, dots below the diagonal). For 13 complexes, AFM produces an MMscore>0.75 and AFP does not but for 50 complexes AFP produces successful complexes when AFM does not. Importantly, the AFM complexes can’t be selected as these all have an RC<0.75. The median MMscore improves with AFP as well (0.562 vs 0.536 with AFM).

### Structures with larger changes are more successful

**Fig 3:**
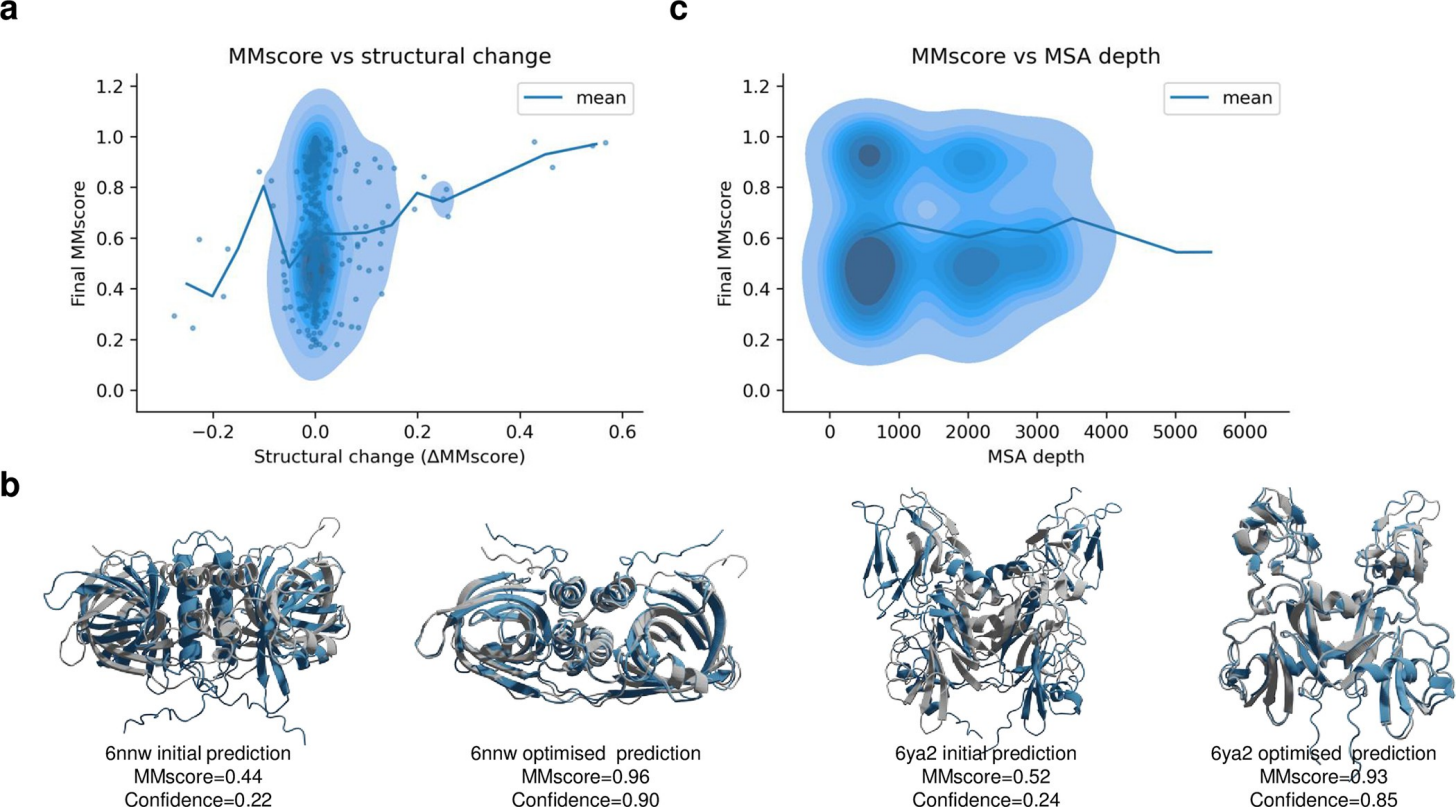
**a)** Final MMscore vs structural change during the optimisation procedure as measured by the change in MMscore (ΔMMscore). The density and points represent all predictions (n = 487) and the line is the running mean with a step size of 0.05 ΔMMscore. This shows that the MMscore is unlikely to decrease with the optimisation procedure and that when the structural change is large compared to the initial prediction (measured by ΔMMscore), it is likely to obtain a higher MMscore. This is expected as the predicted structures are inaccurate using AFM (MMscore<0.75). **b)** Initial and AFProfile optimised structure of PDBIDs 6nnw (https://www.rcsb.org/structure/6NNW) and 6ya2 (https://www.rcsb.org/structure/6ya2). The native structure is in grey and the predicted ones are in blue. The MMscores increased from 0.44→0.96 and 0.52→0.93 and the confidences from 0.22→0.90 and 0.24→0.85, respectively, during the optimisation. **c)** Number of sequences in the MSA input representation generated by the AFM pipeline (MSA depth) vs the final MMscore after optimization with AFprofile. The density represents all predictions (n = 487) and the line is the running mean with a step size of 500 in MSA depth.

As most complexes reached confidence of >0.8, but their MMscores did not improve to above 0.75 (Fig 2A), AFM may not be able to predict these at all (i.e. these targets are too difficult). It is also possible that an alternative conformation is predicted that is currently unknown but this can not be verified. Fig 3A shows the final MMscore vs the structural change during the optimisation procedure with AFprofile as measured by the change in MMscore (ΔMMscore). In most cases, the change in MMscore is small. Even in cases where this change is small, it is important to select the correct models from the confidence scores. It is unlikely to observe a substantial decrease in the MMscore with the optimisation procedure suggesting that when a high MMscore is obtained initially, a similar prediction is maintained.

When the structural change is large compared to the initial prediction it is more likely to obtain a higher MMscore. This suggests that the initial conformations generated by AFM are incorrect in these cases and that AFProfile recovers other conformations that are more likely to be correct. The relationship between the structural change and MMscore is expected as the predicted structures from AFM are likely inaccurate (RC<0.75). Examples for the PDBIDs 6nnw (https://www.rcsb.org/structure/6NNW) and 6ya2 (https://www.rcsb.org/structure/6ya2) are shown in Fig 3B.

In addition, we analysed the impact of the MSA depth (the number of sequences in the input MSA representation from AFM) on the outcome (Fig 3C). The MSA depth is the result of combining single-chain MSAs on the species level, based on gene distance and certain block diagonalisation to retain single-chain information [7]. It does not seem favourable to have any typical MSA depth. Likely, the quality of the information contained inside the MSA and not its size determines the outcome. This may explain why these structures can’t be predicted with sufficient accuracy as well. It may be that the MSAs do not contain sufficient information and, therefore, there is nothing for AFProfile to improve upon.

## Discussion

AFProfile can enhance the outcome of structure prediction with AlphaFold-multimer (AFM) by denoising the MSA profile. The predicted confidence metric from AFM acts as a guide for this process and correlates with the MMscore of the complexes (SpearmanR = 0.59 for the 7 CASP15 targets, Fig 1). Applying AFP to a large test set of 487 complexes where AFM has low ranking confidence (RC, <0.75), results in accurate predictions (MMscore>0.75 and RC>0.8) for 33% of the complexes. However, the amount of hidden failures (RC is >0.8 and the MMscore<0.75) is 200/429, suggesting caution when interpreting the RC. This suggests that AFProfile can improve structure prediction for difficult targets, but the risk of FPs is high.

Using default AFM with thousands of samples may result in similar or better performance. However, the outcome will be uncertain. AFProfile provides a way to direct predictions towards a defined target function guided by the MSA. We expect the procedure of using gradient descent over the MSA to be useful for a variety of tasks such as generating alternative conformations but this first has to be investigated.

## Methods

### Data

#### CASP15

To see if we can improve upon AlphaFold-multimer (AFM) and AFsample, we select eight targets from CASP15 where AFsample significantly outperforms AFM. These are H1134, T1123o, H1129, T1173o, H1141, H1144, H1140 and T1187o. For H1134, the MMscore is not significantly improved, but the interface as measured by the DockQ score [22] is. The suffices (o) from T1123o, T1173o and T1187o are excluded from the referrals throughout the rest of the text. H1129 was disregarded as it was out of memory using an NVIDIA A100 GPU with 40 GB of RAM, resulting in seven complexes in total. We used the same MSAs as AFsample (for consistency) and no template information. We only ran model_1 from AFM v2.0 and not all 5 models available from AFM. The MSAs from AFsample are available through: http://bioinfo.ifm.liu.se/casp15/. The MMscores for AFsample and AFM in Table 1 were obtained from https://predictioncenter.org/casp15.

The best models from CASP15 were used as references as these are all highly accurate and the real structures are not available yet. Alternative conformations may be present here, as the first and second-ranked models from AFsample display completely different binding sites (e.g. H1144, S1 Text) [9].

### AlphaFold-multimer benchmark set with 2–6 chains

It is necessary to have an evaluation set of sufficient size for multimers. Therefore, we use a set from a recent benchmark of AlphaFold-multimer (AFM) of 2–6 chains that have been homology reduced on the structural level with pairwise MMscores<0.6 [3]. There, AFM v2.2.0 was run with default settings (including templates and 5 models per model weights, 25 models in total) and the top-ranked model was selected for the analysis.

We use AFM v2.2 for comparison as v2.3 has a cutoff date of 2021-09-30 and v2.2 of 2018-04-30, leading to much fewer structures being available for evaluation with v3. In addition, the improved models in CASP15 from AFsample all came from AFM v2 [9,17]. We selected the examples which have ranking confidence <0.75 (Eq 1) [3] as these can be selected from a single AFM run, to see if we can improve the structural accuracy and the possibility of selecting accurate models.

Of the 1928 complexes, 1011 have a ranking confidence <0.75. Of these, 648 were unsuccessful (DockQ score <0.23 [22] and an MMscore <0.75 [2]) and 363 were successful (36%). This suggests that some predictions are accurate, but AFM can’t select them. From the 1011 complexes, 427 (42%) could be predicted using an NVIDIA A100 GPU with 40 GB RAM within an 8-hour time limit. AFProfile requires more RAM than AFM due to the gradient calculations, resulting in the largest complexes do not fit within the 40 Gb limit. For AFProfile, the models with the highest confidence were selected and scored in all analyses.

### AlphaFold-multimer v2

To predict the structures, we used AlphaFold-multimer (AFM)[7] v2 and the parameters from 2022.03.02 available from: https://storage.googleapis.com/alphafold/alphafold_params_2022-03-02.tar. We did not use any template information and turned on dropout to introduce noise everywhere except for in the structural module [9]. We only used one set of weights (model_1) from the five available [7] and used the default MSA generation pipeline (below).

By searching various databases with several genetic search programs, multiple sequence alignments (MSAs, four in total) are created. Jackhmmer from HMMER3 [23] is used to create three different MSAs by searching the databases Uniref90 v.2020_01 [24], Uniprot v.2021_04 [25] and MGnify v.2018_12 [26]. By searching the Big Fantastic Database [27] (BFD from https://bfd.mmseqs.com/) and uniclust30_2018_08 [28] together with HHBlits [29] (from hh-suite v.3.0-beta.3 version 14/07/2017) the fourth MSA is created. We used the reduced BFD option to save time and space. Species and positional information of genes are used to pair the results from the Uniprot search. The results from the other searches are block-diagonalized to maintain as much intra-chain information as possible. All four MSAs are used for the protein complex prediction by sampling and clustering the information.

### Finding the best profile residuals

The intuition behind directing the prediction process with a profile residual is that a random sample results in a structure that is accurate locally (i.e. for each single chain) but may not be so globally (the entire complex). The problem is to traverse the conformational landscape to the lowest valley, i.e. the best score [8,11]. The rationale we use is that AFM is capable of generating highly accurate predictions for all targets given informative sequences, but these have to be found. Another aspect is that the network needs to build up a sufficiently large internal representation of information. This is maintained by recycling and by resampling the MSA during each recycle. The MSA cluster profile bias is added in each recycle, which helps to direct the internal information in a more favourable direction.

One could sample diverse sequences randomly, but the many possible combinations make the probability of finding the right ones unlikely. For this reason, many samples are drawn by AFsample. The most efficient way to direct the internal representation should be with gradient descent, using the fact that the network has learned the relationship between the MSA profile and the structure. One could traverse the MSA profile directly to generate a structure, but this may introduce issues between iterations due to different sequences being sampled. Therefore, we generate a bias to the MSA profile that learns to be robust across different samples and profiles.

We only use the first model (“model_1”) generated by AFM v2, although 5 models are available. The other four models are not explored to reduce computational cost and the fact that the weights should be highly similar and respond similarly to similar MSA profiles. We use a maximum of 100 iterations and Nvidia A100 GPUs with 40 GB RAM. The complexes that did not fit within these limitations were disregarded.

We explored varying the number of recycles between 0, 5, 10, 15 and 20 and using different learning rates (1e-4, 1e-3, 1e-2) with the Adam optimiser [30]. Note that only the gradients at the final pass through the network are used since we can’t take the gradients across recycles. This is consistent with the training procedure of AFM where only the gradients of the last recycle operation are used. The loss function used for all settings is confidence^-1^, where the confidence is defined as in Eq 1.

**Fig 4:**
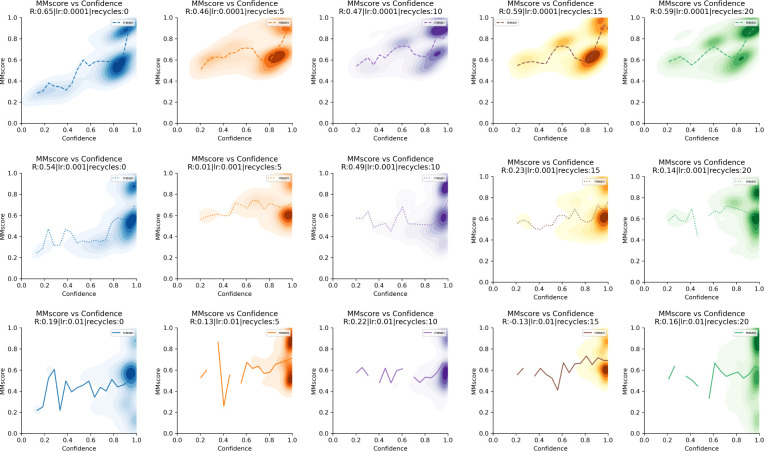
MMscore vs confidence for all 7 complexes using 100 iterations per complex (n = 700) from the CASP15 set. The learning rate (lr) is increasing row-wise and the number of recycles column-wise. The Spearman correlations (R) are displayed in the figure legend. Some correlations are negative. The best correlation (0.68) is obtained by using lr = 0.0001 and 0 recycles.

It is not only important to obtain fast convergence, but also to obtain high scores. To analyse the relationship between confidence and model accuracy we used the MMscore from MMalign [2]. We use the best predictions from the CASP15 (Table 1) as the reference structures. The relationship between the MMscore and ranking confidence is noisy and in some cases, it is decreasing (S1 Text). For most targets, the MMscore increases with the ranking confidence (S1 Text). The best Spearman correlation (0.65) is obtained by using a learning rate of 0.0001 and 0 recycles. However, a lr of 0.0001 and 20 recycles results in a higher density of highly accurate predictions at high confidences with a correlation of 0.59 which is why this is preferred (Fig 4).

The correlations tend to decrease with an increasing learning rate, suggesting that it is not beneficial to traverse the loss landscape in too large steps but rather to approach the highest confidence levels slowly. This is true for all targets except for H1141 where the optimisation procedure seems to get stuck at MMscores of 0.7 using a lr 0.0001 and a higher learning rate (0.01) results in MMscores>0.95.

In all analyses with AFProfile, the models with the highest confidence were selected and scored as the top models.

### Description of the AFProfile algorithm

Here we describe AFProfile in detail. Starting from a sequence, the default input feature generation pipeline from AFM is used except for not using any templates (see AlphaFold-multimer v2 section). Several input feature representations are generated, but the main focus is on the resulting MSA cluster profile, which is part of the MSA representation [7]. We introduce a bias to the cluster profile with an identical shape to this that is modified at each gradient update:

1. Initialise a bias to the MSA cluster profile (zeros with the same shape as the profile).
2. Pass the bias as another input to the prediction pipeline and add this to the MSA cluster profile.
3. Run a forward pass through the network to obtain a predicted confidence score (Eq 1).
4. Take the gradients over the resulting confidence score as a function of the bias.
5. Update the bias according to the gradients to maximise the confidence (minimise 1/confidence).

The Adam optimiser [30] with a learning rate of 0.0001 and a maximum of 100 steps is used. To make the bias robust to sampling noise, dropout is activated everywhere except in the structural module [9]. The number of recycling operations used is 20 and these are performed within each forward pass. This means that the recycled information is not carried over between forward passes and the bias term has to act across different MSA samples and recycles. The learned bias is also not transferable but specific for each protein complex. For each complex that is to be predicted, a new bias has to be generated by gradient descent.

### Scoring

We used MMscore from MMalign [2] to score the predicted complexes. This score has a high Spearman correlation with the DockQ score [21] in the dataset we use for evaluation here (>0.8 for heteromers and >0.9 for homomers [3]). MMscore in addition captures alternative positions of identical chains (homomers), which DockQ does not, making it a better score for evaluation as such “swaps” should result in identical scores.

For the CASP15 structures, we can’t compare them with the correct models as these are not available. However, the structures we selected for development (the highest scoring structures, Table 1) all report MMscores >0.9 and should therefore serve as adequate substitutes.

## Supporting information

S1 Text Supplementary figures. Supplementary notes.
(PDF)
