# Supplementary material for: Improved protein complex prediction with AlphaFold-multimer by denoising the MSA profile
Source: PLoS Comput Biol. 2024 Jul 25;20(7):e1012253. doi: 10.1371/journal.pcbi.1012253 (PMC11302914; doi:10.1371/journal.pcbi.1012253)
Supplement: S1 Text — Supplementary notes. (PDF) [file pcbi.1012253.s001.pdf]

Supplementary material

Supplementary figures

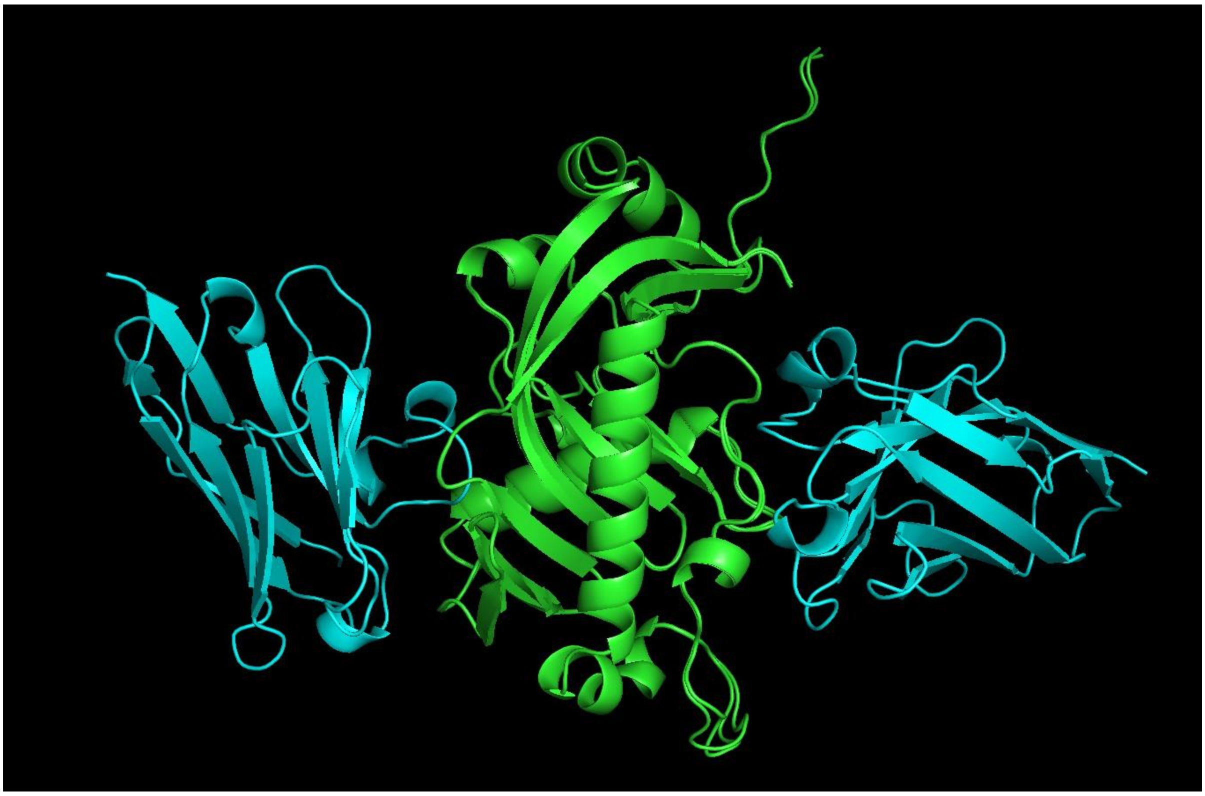

Supplementary Figure 1. First (blue chain to the left) and second (blue chain to the right) ranked models superposed on the green chain from AFsample for target H1144.

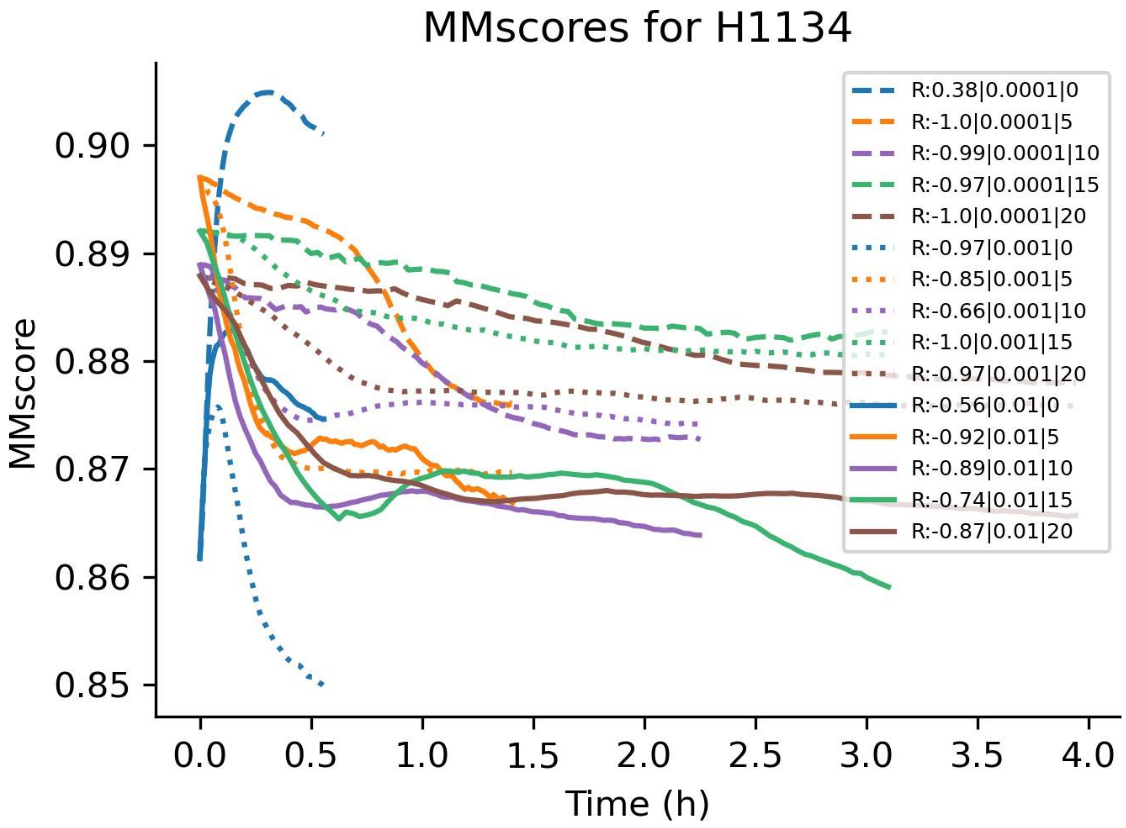

Supplementary Figure 2. MMscore vs time for H1134 using different learning rates and recycles. The Spearman correlations (R) are displayed in the figure legend. The MMscore is stable when a good starting position is found for lower learning rates.

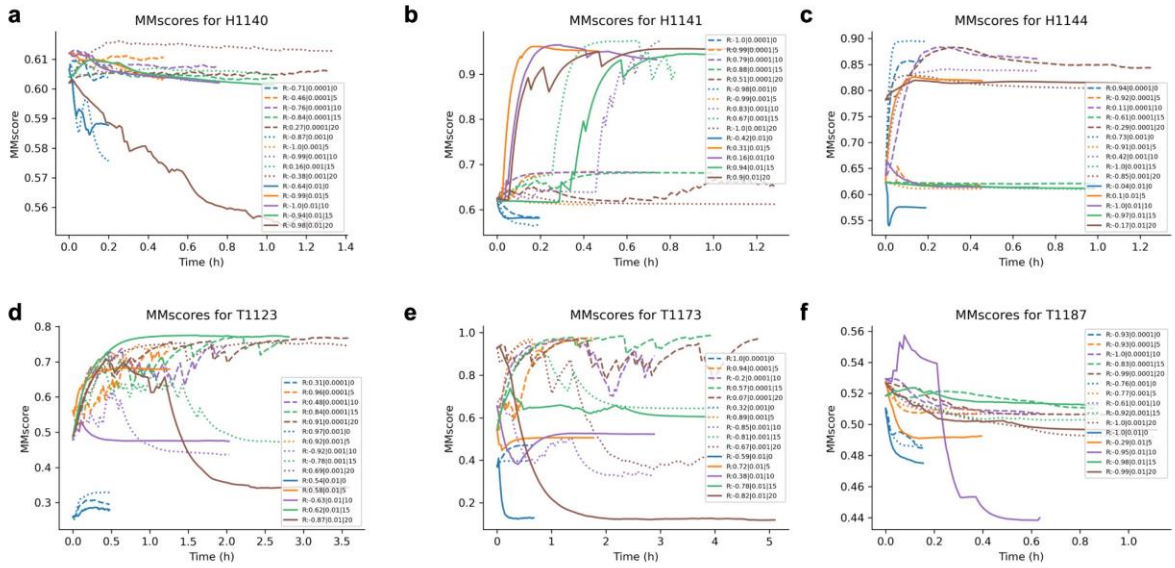

Supplementary Figure 3. MMscore vs time for the targets H1141, H1144, H1140, T1123, T1173 and T1187 (a-e). The Spearman correlations (R) are displayed in the figure legend.

The MMscore increases with time and thereby confidence for some settings in all cases except for T1187.

Supplementary note 1

To estimate how useful the ranking confidence (RC, equation 1) is for AFProfile to select accurate models, we create a ROC curve. AFProfile obtains a ROC AUC of 0.74 in selecting accurate models (MMscore>0.75) using the RC . The AUC is 0.74 compared to 0.5 for a random selection.

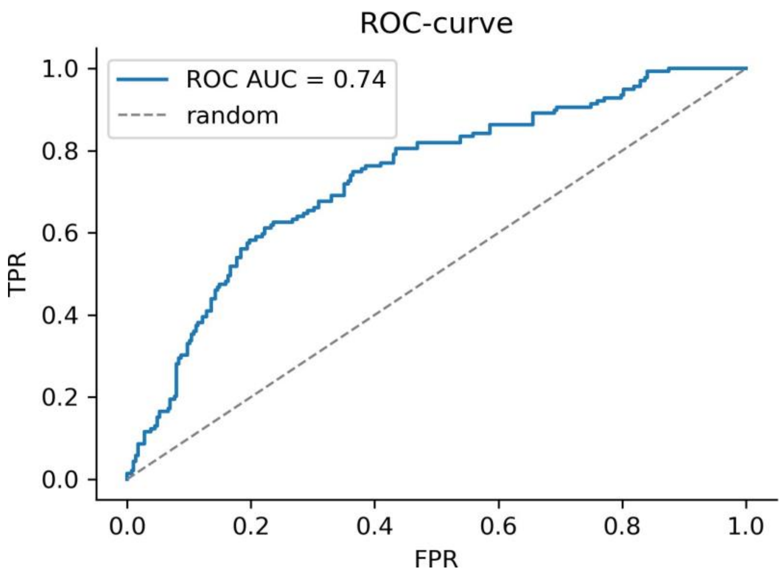

Supplementary Figure 4. ROC-curve (FPR vs TPR) for AFProfile in selecting accurate models (MMscore>0.75) using the ranking confidence *(equation 1)*. The AUC is 0.74 compared to 0.5 for a random selection.

Supplementary note 2

Instead of using MMalign, USalign can also be used. We ran USalign (https://github.com/pylelab/USalign) for the top-scoring models in Figure 2 (n=487). It is evident the scores are very similar (Pearson correlation coefficient = 0.996), although USalign provides higher scores in some cases

We ran USalign with the following options:

$USALIGN -mm 1 -ter 0 $PRED $NATIVE

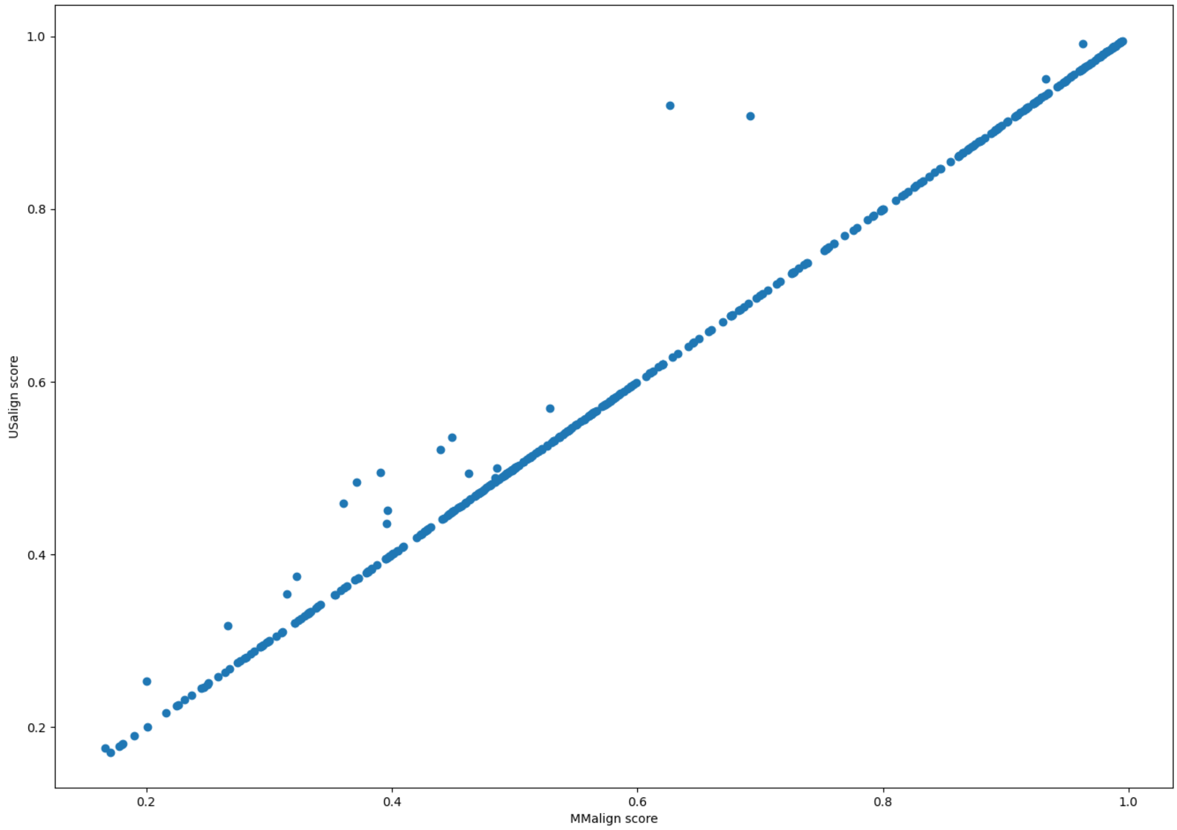

Supplementary Figure 4. Comparison of MMalign and USalign TM-scores. In no case does MMalign give a higher score, meaning that this score is more stringent. The Pearson correlation coefficient is 0.996.
